# Barriers and facilitators to type 2 diabetes management among slum‐dwellers: A systematic review and qualitative meta‐synthesis

**DOI:** 10.1002/hsr2.1231

**Published:** 2023-04-27

**Authors:** Fawzieh Ghammari, Habib Jalilian, Rahim Khodayari‐zarnaq, Masumeh Gholizadeh

**Affiliations:** ^1^ Department of Health Policy and Management, School of Management and Medical Informatics Tabriz University of Medical Sciences Tabriz Iran; ^2^ Department of Health Services Management, School of Health Ahvaz Jundishapur University of Medical Sciences Ahvaz Iran

**Keywords:** barriers, diabetes management, disadvantaged groups, facilitators, slums, T2D

## Abstract

**Background and Aims:**

The prevalence of type 2 diabetes (T2D) is on the rise worldwide, especially in developing countries. There is a significant difference between the slum‐dwellers and other urban dwellers in terms of T2D incidence rate and access to healthcare services. This review aimed to identify barriers and facilitators to T2D management among slum‐dwellers.

**Methods:**

A systematic review was conducted to identify barriers and facilitators to T2D management from January 1, 2002 to May 30, 2022. We searched MEDLINE via PubMed, Scopus, Web of Sciences, and Google Scholar. The inclusion criteria were: qualitative or mixed‐methods research, published in English, focused on slum‐dwellers and T2D or its complications, and assessed barriers and facilitators to T2D management among slum‐dwellers. Quality appraisal was conducted using the QATSDD critical appraisal tool. A thematic approach was used for data analysis and synthesis.

**Results:**

A total of 17 articles were included in this review. Three analytical themes were identified: (1) Individual factors consisting of four themes: lifestyle behaviors, informational, psychological, and financial factors; (2) Health system factors consisting of three themes: patient education processes, financial protection, and service delivery; and (3) Contextual factors consisting of three themes: family support, social support, and environmental factors.

**Conclusion:**

Our review disclosed that the individual, health system, and context influence T2D management among slum‐dwellers. Policymakers can use the findings of this review to reduce barriers and augment facilitators to improve healthcare utilization and self‐care management among patients with T2D in slums.

## 1 INTRODUCTION

Slum‐dwellers are socioeconomically and environmentally disadvantaged.^1^ According to the United Nations Human Settlements Program, in low‐ and middle‐income countries (LMICs), nearly 863 million people are estimated to be living in slums.^2^ Slums are an important part of today's urban settlements.^3^, ^4^ The United Nation Program on Human Settlement has defined slums in 2002 as: “a contiguous settlement where the inhabitants are characterized as having inadequate housing and basic services. A slum is often not recognized and addressed by the public authorities as an integral or equal part of the city.”^5^ In detail, a slum is an urban area characterized by poor health outcomes, low access to health services, low housing situation, poor social services, inadequate basic facilities, insecurity, poor livelihood, unstable incomes, overcrowding, poor sanitation infrastructure, and limited access to safe water.^3^, ^4^, ^6^

Type 2 diabetes (T2D) is among the most prevalent noncommunicable diseases (NCDs) globally.^7^ The total number of people living with diabetes is predicted to rise to 643 million by 2030 and 783 million by 2045.^8^ A total of 75% of diabetic patients live in developing countries. The prevalence, risk factors, and complications of T2D are higher among the disadvantaged population,^9^ including slum‐dwellers.

A study in India demonstrated that the prevalence of people at high risk for diabetes was high in the slum population, and primary education, low socioeconomic status, less physical activity, and high waist circumference were major contributing factors to diabetes.^10^ Also, a study in Brazil showed the prevalence of T2D was higher among slum‐dwellers compared to the general population (10.1% vs. 5.2%).^11^

Despite the high level of healthcare needs, slum‐dwellers are less likely to seek and use healthcare services than the nonslum population in the cities.^12^, ^13^ For instance, it has been shown that slum‐dwellers have lower healthcare utilization in antenatal services^12^ and also NCDs services^13^ compared with the general population. Given that slum‐dwellers are a population with specific needs and are at higher risk for the incidence of T2D and its complications, the management of T2D among this population should be taken into account specifically. Slum‐dwellers face more challenges and barriers to better management of T2D. Identifying barriers and facilitators to T2D management can assist policymakers in augmenting facilitators and modifying barriers to improve healthcare utilization among different communities of slum‐dwellers. This review aimed to comprehensively identify T2D management barriers and facilitators among slum‐dwellers.

## 2 METHODS

### 2.1 Data sources and search strategy

To conducting this systematic review, we searched MEDLINE via PubMed, Scopus, and Web of Sciences from January 1, 2002 to May 30, 2022 using the following keywords: facilitators, barriers, management, health‐seeking, adherence, compliance, utilization, T2D, and slum‐dwellers. To conduct a comprehensive search, synonymous terms were found using mesh terms. Google Scholar was searched for relevant articles. Gray literature was also searched. In addition, we searched the reference lists of all included studies to identify additional relevant studies. The full search strategy and keywords can be found in Appendix 1.

### 2.2 Eligibility criteria

The articles were included if they (1) published between January 1, 2002 (slum‐dwellers was defined based on the United Nations) and May 30, 2022, (2) focused on slum‐dwellers, (3) focused on T2D or its complications, (4) used a qualitative approach or mix‐method approach, and (5) examined barriers or/and facilitators to T2D management in slum‐dwellers. Articles that were not published in the English language were excluded.

### 2.3 Study selection

All duplicate articles were removed using the EndNote reference management software, and two independent reviewers (F. G. and H. J.) reviewed the titles and abstracts of retrieved articles for eligibility. After removing the irrelevant articles, the full‐text studies were independently assessed for eligibility by the two researchers. In the case of disagreement in the selection process, each disagreement was resolved by discussion with the third independent researcher.

### 2.4 Data extraction

Once the article was deemed to be eligible, data were extracted independently and summarized by two researchers (F. G. and M. G.) and checked by a third reviewer (H. J.). A standard form was developed for data extraction. Extracted details included author (year), location, quality assessment, objective/s, design, sample size (participants), data collection methods, age and sex, literacy level, race barriers, and facilitators (Tables 1 and 2). We defined barriers as any factors that their absent/presence impede slum‐dwellers from managing their diabetes properly. In our definition of facilitators, we refer to factors that their presence promotes the management of T2D.

**Table 1:** The characteristics of included studies and their participants.

References	Location	Quality assessment	Objective/s	Design, sample size (participants), and data collection methods	Age and sex	Literacy level	Race
1) Vest et al.^14^	The west side of Buffalo, New York, USA	Moderate	Exploring the role of social assistance and institutional resources on diabetes self‐management	Qualitative, 34, semi‐structured interviews	Mean: 58 ± 11 years Male (26) Female (8)	≤8th grade (14) Some high school (5) High school graduate (10) Some school graduate (2) College (1)	Africa (6) Asia (5) Latino (11) Non‐Hispanic White (6) African American (4) Native American (2)
2) Power et al.^15^	New South Wales, Australia	High	Explaining T2D^a^ patients' experiences	Qualitative, 17, semi‐structured interviews, face to face	Range 21–74 years Male (9) Female (8)	Not mentioned	Aboriginal (8) Samoan (2) German (1) Swedish (1) White Australian (5)
3) Ponaiah et al.^16^	Tamil Nadu, India	High	Exploring older women's experiences with self‐care management and challenges of T2D	Qualitative, 22, focus group discussion	Range 60–75 years Female (22)	Primary education (3) High school (6) Senior high school (13)	Asian
4**)** Mphwanthe et al.^17^	Kasungu, Malawi, Southeastern Africa	Moderate	Exploring barriers, facilitators, and support for exercise and diet	Qualitative, 18, focus groups	≥40 years, Mean: 53.1 ± 7.5 years Male (8) Female (10)	≤primary level (77.8) ≥Secondary level (27.8)	African
5) Mayega et al.^18^	Iganga, Uganda, East Africa	High	Assessing perceptions and lifestyle changes among T2D patients or high‐risk individuals of it	Qualitative, 96, focus groups	Range 35–60 years Male (50%) Female (50%)	None (20%) Lower primary (21%) Higher primary (36%) Secondary (18%) Tertiary (0.05)	African
6) Masupe et al.^19^	Cape Town, South Africa	Moderate	Understanding contextual and environmental factors influencing care and self‐management	Qualitative, seven focus groups (56), two in‐depth interviews, in‐depth interviews, and focus groups	Range 30–70 years. There is no mention of the gender of participants in the number	Not mentioned	African
7) Leyns et al.^20^	Cochabamba, Bolivia	Moderate	Exploring perceived needs, actual needs, and needs modifying	Qualitative, 33, highly structured qualitative brainstorming	Range 41–80 years Male (4) Female (25)	No education (9) Primary education (13) Secondary education (9) College/university (2)	Not mentioned
8) Cuesta‐Briand et al.^21^	Perth, Australia	Moderate	Exploring the impact of indigenous status and socioeconomic disadvantage on the experience of diabetes care	Qualitative, 38, focus groups and interviews	Range 25– >75 years Male (10) Female (28)	Years 8–12 (26) TAFE (7) Bachelor's degree (7) Data not available for the rest of the participant	Australian (29) Other (9)
9) Bhojani et al.^22^	KG hall, Bengaluru, India	Moderate	Barriers to care‐management	Qualitative, 16, in‐depth interviews	Range 21–65years Male (9), female (7)	Not mentioned	Asian
10) Nimesh et al.^23^	Bhopal, India	Moderate	Explore health‐seeking patterns, provider preferences, and switching reasons	Mixed‐method, 60, semi‐structured questionnaire, in‐depth interview	≥20 years, mean: 52.35 ± 9.07 years Male (28), female (32)	Illiterate (21) Literate (39)	Asian
11) Jackson et al.^24^	Memphis‐Whitehaven, Tennessee, USA	Moderate	Identifying unmet primary care needs	Community case study, 5723, interviews (9), focus groups (11), multiple methods and data sources: Registry data, interviews, focus groups, and a cross‐sectional survey	Interviews: Range 22–78 years, 8 female, 1 male Focus group: range 40–70 years, 9 female, 2 male	Not specified for qualitative study	African American non‐Hispanic
12) Baghikar et al.^25^	South Lawndale, Chicago, USA	Moderate	Identifying barriers and facilitators to treatment adherence	Qualitative, 27, semi‐structured interviews	Mean 57 ± 11 years Male (5) Female (22)	Less than high school diploma (19)	Latino
13) Gazmararian et al.^26^	Atlanta, Georgia, USA	Moderate	Identifying personal, educational, and system barriers to optimal self‐management	Qualitative, 35, 3 focus group discussions including 11 new patients, 12 current patients, 11 infrequent patients	New patients (average 48 years), current patients (average 58 years), infrequent patients (average 54 years) There is no mention of the gender of participants in the number	Less than high school (22.8) High school (25.7) Some college, trade, or tech school (0.42) College graduate (0.08)	African American (0.88) White (0.08) Latino (0.02)
14) Rose et al.^27^	Southwest Sydney region, Australia	Moderate	Exploring GP^b^ views about the management of T2D patients	Qualitative, 9, focus group	Range 45–64 years Male (7) Female (2)	General practition	Asian
15) Onwudiwe et al.^28^	Baltimore, USA	Moderate	Exploring patients' perceptions about diabetes self‐management barriers and possible explaining for poor health outcomes in minority patients	Qualitative, 31, focus group interviews	Range 43–81 years There is no mention of the gender of participants in the number	Not mentioned	African American
16) Hu et al.^29^	North Carolina, USA	High	Exploring perceived barriers in Hispanic immigrant diabetic patients and their family members	Qualitative, 73, focus group interviews	Patients (mean: 50 ± 10.77 years) Family member/significant others (mean: 41 ± 13.12) Male (0.25) Female (0.075)	Not mentioned	Hispanic
17) Tiedt et al.^30^	Coeur d'Alene tribe, Columbia Plateau region, USA	Moderate	Identifying barriers to diabetes self‐management in tribal patients	Qualitative, 10 in‐depth interviews	Range 26–86 years Male (3) Female (7)	Not mentioned	Native American

^a^ Type 2 diabetes.

^b^ General practitioner.

**Table 2:** Identified barriers and facilitators.

**Study author**	**Barriers**	**Facilitators**
Vest et al.	Fear of becoming a burden on family Fear of losing independence Lack of trust in healthcare providers Lacking or inadequate insurance	Assisting in the instrumental day‐to‐day care Moral/emotional support Children as motivators Access to informal medical information Trust in physicians
Power et al.	Wrong perceptions about diabetes and its causes Forgetting to use medicines Stigma and self‐blame inertia Illiteracy Comorbidity Financial constraints Access issues Transportation issues Being unsafe in the area Insufficient services Dissatisfaction with the presented information Healthcare providers are unhelpful and judge patients Unpractical advice from dieticians Impersonal consultations	Care delivery in the community Providing/presenting information in the community Transport improvement Narrative information and practical programs
Ponaiah et al.	Difficulty adhering to the diet and doing physical activity regularly Psychological issues Fear of medicine complications	Online group discussion
Mphwanthe et al.	Cost Information Lack of access to an appropriate dietary Perceived barriers to exercise are comorbidities Fear of public ridicule Family size	Socio‐support system to provide dietary and exercise Diabetes peer groups Family member support
Mayega et al.	Perceptions about the severity of diabetes Lifestyle change is associated with some risks Lifestyle change is a hurdle to a good life Poverty Unavailability of services Family size Lack of access to some food is a barrier to appropriate dietary adherence	The exercise is possible just in familiar gatherings
Masupe et al.	Unavailability of health services Lack of information and awareness about diabetes and its severity Lack of support from the healthcare provider Self‐acceptance and denial, stigma in some cultures Individual options	Awareness about the causes of diabetes Understanding and informing about healthy diet and exercise importance Traditional and modern interventions
Leyns et al.	Poor literacy Linguistic constraints Lack of support from the healthcare provider	Information about appropriate management of diabetes Access to health services and competent healthcare providers Availability of health services and health resources, and insurance Healthy food availability Safety area Family and community participation
Cuesta‐Briand et al.	Very limited access to dietitians and podiatrists Perceived need Cost Poor information about available services Prior negative experiences Poor coordination of care	Regular access to GPs^a^
Bhojani et al.	Financial issues Negative attitude and poor communication with healthcare providers Poor services provided by the fragmented health system The influence of sociodemographics is the most reason for poor access to healthcare	A good relationship with some physicians
Nimesh et al.	Financial barriers Transportation issues Limited access to medicines Medicine complications Long waiting time to receive a consultation Difficulty adhering to the diet	Offering free services by the healthcare provider Trust in healthcare provider Healthcare provider competencies Satisfaction with the healthcare provider
Jackson et al.	Inaccessibility to healthcare Insufficient coverage Poor relationship with a healthcare provider Poor primary care and social support and resources for diabetes care	Information about diabetes and its management A good relationship with a healthcare provider Support for problems arising from diabetes Focus group Healthcare access and coverage Support and resources for dealing with diabetes Support for overcoming health literacy barriers and achieving diabetes goals
Baghikar et al.	Beliefs about unnecessary taking medication due to complications of medications and ineffective medication use Poor relationship with the healthcare provider	Understanding the importance of the use of medicine Family support Follow up to use the medication by the healthcare provider Taking medication as recommended to maintain good health status Public health interventions to educate patients on diabetes self‐management need
Gazmararian et al.	Individual barriers (emotional complications) Educational barriers (unknowing of complications of asymptomatic diseases) System barriers (needed services)	Follow‐up Providing regular educational courses Communication Education about dietary and treatment Diversity of education Increased clinic hours
Rose et al.	Poor health literacy Poverty Psychological concerns Concerns about services available A negative attitude toward health	Creating educational materials tailored to patients' literacy level Providing financial incentives
Onwudiwe et al.	Attitude, perceptions, and behaviors about diabetes and self‐management are different and depend on persons' age, gender, and culture The lack of knowledge about target blood glucose and blood pressure	Not mentioned
Hu et al.	Enduring diabetes Difficulty in diabetes management Poor resource/support Support provision Lack of knowledge of family members about the disease A lack of financial resources	Not mentioned
Tiedt et al.	Relationships (mistrust, bad understanding, and methods of training) and organizational (care quality and problems to access) barriers as disappointing care to self‐management	Holding meetings in the community to determine preferred learning styles, content, and recruitment methods for diabetes self‐management educational sessions The educational approach to storytelling Talking circles, hands‐on demonstrations, culturally based videos and visual aids, classes, and resources embedded in the community Building trust and enhancing communication between patients and providers

^a^ General practitioner.

### 2.5 Quality assessment

Two reviewers (F. G. and H. J.) appraised each of the included studies using the Quality assessment tool for studies with Diverse Designs (QATSDD).^31^ The QATSDD consists of 16 items scored on a four‐point Likert scale, ranging from 0 to 3. Some items are just about assessing qualitative or quantitative studies. The maximum score that could be obtained was 48 for mixed‐ and multimethods and 39 for qualitative studies. After normalizing the obtained score of each study, the studies were categorized into three categories; studies scoring between 81 and 100 were considered “high quality,” 60 and 80 “moderate quality,” and those scoring 59 or below “poor quality” (for more details, see Appendix 2).

### 2.6 Analysis and synthesis

We used qualitative thematic synthesis for data analysis, as proposed by Thomas and Harden.^32^ This thematic synthesis analysis has three stages^1^: the coding of text “line‐by‐line,”^2^ the development of “descriptive themes,” and^3^ the generation of “analytical themes.” We first used an inductive approach to coding and to create new themes and then complimented the process with a deductive approach to categorize codes. In this review, stages 1 and 2 were concurrently conducted by two reviewers. In the development of the primary synthesis, two independent reviewers (F. G. and M. G.) undertook a line‐by‐line review of each study's results and discussion and extracted initial codes. Coding was based on explicit and implicit responses to the study question. Next, identified codes were compared based on their similarities and differences. After the agreement between reviewers about codes, similar codes were grouped, and descriptive themes were developed accordingly. The name of each descriptive theme was chosen with the agreement between reviewers and derived based on the most common meaning conveyed by the codes across studies. This iterative process was continued until saturation of themes and codes and agreement was achieved between reviewers. After that, the themes were examined in terms of similarity and differences, and the similar descriptive themes were categorized in an analytical theme. To develop analytical themes, we generated new interpretive constructions beyond the primary studies. The analytical themes were developed by two independent reviewers. Then, decision‐making about analytical themes was discussed among reviewers, and the nomination of analytical themes was discussed among reviewers.

### 2.7 Credibility and trustworthiness

Triangulation and peer debriefing strategies were used to address credibility and trustworthiness.^33^, ^34^ To this end, coding, appraisal, data analysis, and synthesis of studies were conducted by two researchers, independently. Also, discrepancies were resolved through consensus among four researchers. The codes, descriptive themes, and analytical themes were subsequently assessed by two researchers outside of the study.

This systematic review was conducted in accordance with PRISMA 2020 guidelines.

## 3 RESULTS

### 3.1 Study selection

**Figure 1:**
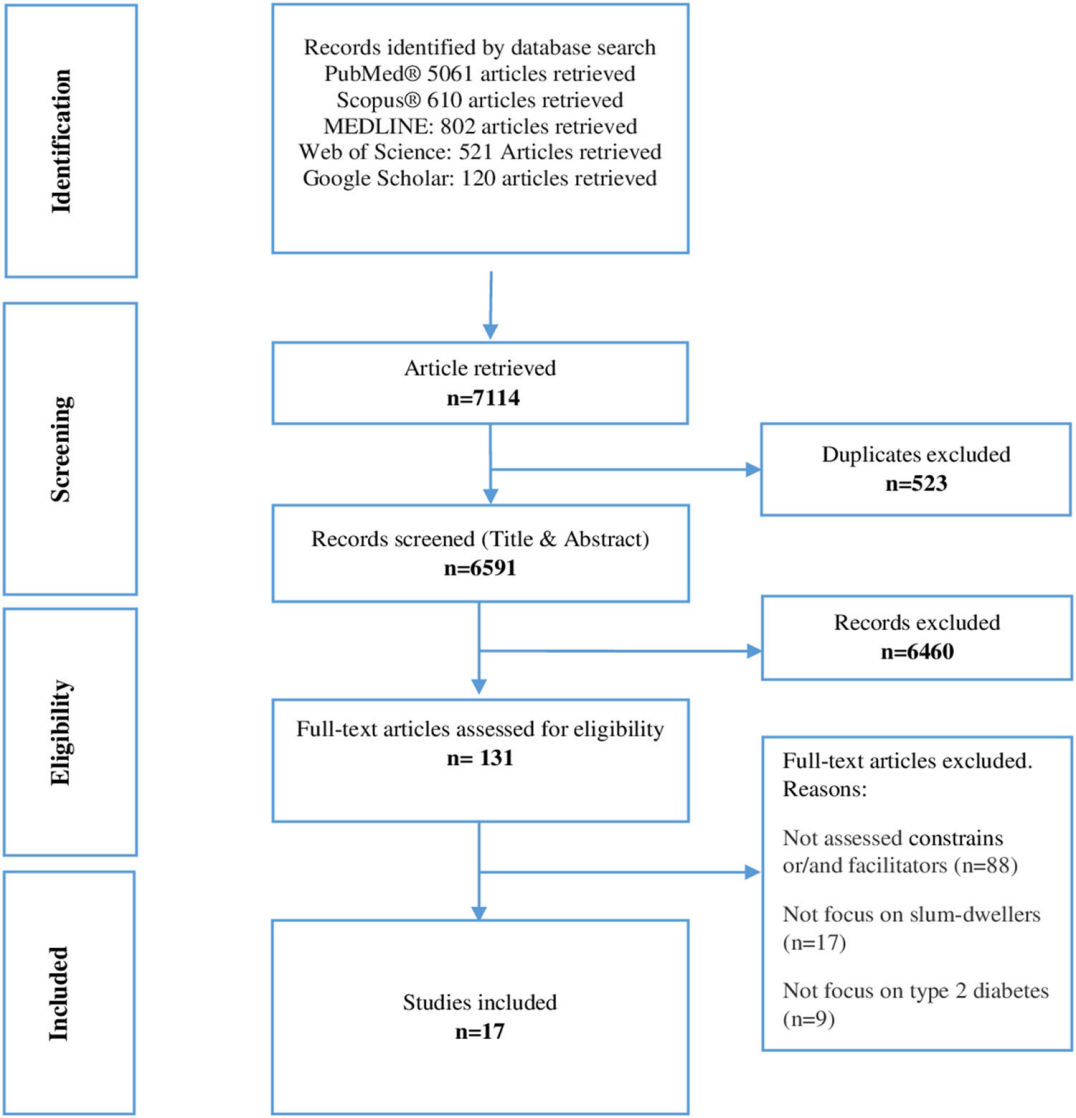
PRISMA 2020 flow diagram of the paper selection process.

As shown in Figure 1, 7114 articles were retrieved from the search strategy. After the removal of duplicates, titles, and abstracts of 5460 articles were screened, and 5329 irrelevant articles were excluded. By assessing the full text of the remaining 131 articles, 107 that did not fulfill inclusion criteria were excluded. Finally, 17 articles met the eligibility criteria and were included in the thematic synthesis.

### 3.2 Study characteristics

The study characteristics of the included studies are summarized in Table 1. The studies were published between 2004 and 2021. Of the included studies, 15 studies were qualitative studies, and two studies were undertaken using mixed‐ or multimethod. Data collection methods for qualitative studies were focus group interviews, semi‐structured interviews, in‐depth interviews, or structured qualitative groups. The sampling methods were purposeful or convenience. Except for one paper, the rest of the studies were conducted from patients' perspective. The quality assessment results showed that four articles had “high quality,” and 13 had “moderate quality.” Most of the included studies were carried out in the USA (*n* = 7), India (*n* = 3), Australia (*n* = 3), South Africa (*n* = 1), Malawi (*n* = 1), Uganda (*n* = 1), and Bolivia (*n* = 1), respectively. Participants of the studies were between 20 and 86 years old. The majority of participants had less than a high school education. In terms of race, the majority of participants are African, Asian, Latino, Native American, African American, and Australian aboriginal. Eight studies^14^, ^16^, ^19^, ^26^, ^27^, ^28^, ^29^, ^30^ reported factors related to diabetes self‐management (four studies reported experiences, challenges, and barriers to self‐management, two studies reported self‐management from the patient's perspective, and two studies from a general practitioner's (GPs) view or healthcare workers,^19^, ^27^ one study reported the disadvantages of living with diabetes,^15^ two studies reported factors impacting adherence to diabetes medication^25^ and barriers and facilitators to diet and physical activity,^17^ one study reported constraints from the patient's perspective,^22^ two studies reported needs and resources^20^ and unmet primary care needs^24^ among patients, one study reported patterns of healthcare‐seeking behavior,^23^ one study reported experiences of patients regarding diabetes medical care,^21^ and one study reported perceptions about severity of type 2.^18^

### 3.3 Barriers and facilitators to T2D management

As shown in Table 3, identified analytical themes were individual factors, healthcare systems and contextual factors. The factors were classified into 10 themes and 38 codes. These factors are often interrelated which can exert their influence at various levels in various circumstances.

1. Individual factors
  Individual factors were approximately reported in all included studies: (1) lifestyle behavior, (2) informational, (3) psychological, and (4) financial.
  1.1 Lifestyle behaviors
    These included change resistance, comorbidities, nature of the disease, lifestyle adherence, and treatment adherence.^15^, ^16^, ^17^, ^18^, ^23^, ^25^, ^26^, ^29^, ^35^ Barriers to lifestyle change were inadequate knowledge of the difference between healthy and unhealthy foods, the cost of buying healthy food, cultural practices, poverty, comorbidities, family size, and access to some foods, losing a job, social isolation, conflicts at home, embarrassment, and worry.^15^, ^17^, ^18^, ^19^, ^26^ Some patients with diabetes expressed that there was an opportunity cost to lifestyle change.^18^ Daily blood glucose monitoring, medication, and lifestyle modifications related to diet, and exercise identified important factors in successful diabetes management.^14^
  1.2 Informational factors
    Four codes on how “informational factors” affect diabetes management were identified in 13 studies^14^, ^15^, ^17^, ^18^, ^19^, ^20^, ^21^, ^24^, ^25^, ^26^, ^27^, ^30^, ^35^: literacy, disease knowledge, knowledge of healthy lifestyle, and health beliefs. Some cultures have a negative perception/attitude toward losing weight and consider it a sign of illness.^15^, ^18^, ^22^, ^25^, ^27^, ^35^ Low socioeconomic position patients had low levels of health literacy,^15^, ^27^ leading them to consume a diet inappropriate for diabetes control.^27^ A study in Australia reported that the perceived need for podiatrists was generally low due to a low level of awareness of patients on the importance of foot care.^21^ Self‐care interventions and Motivational text messages from primary care and health coaching can help meet the healthcare needs of diabetic patients in Medically underserved areas.^24^, ^36^, ^37^, ^38^
  1.3 Psychological factors
    Fear, depression, and denial were barriers to diabetes management in slums. A study in India showed that diabetes in older adults was linked with an escalated risk of psychological disorders and accompanying poor health.^16^ Depression; concerns about the negative impact of diabetes medication and doubts about its effectiveness; stress; frustration; social isolation; interpersonal conflicts; depression; fear and denial; emotional suffering included depression, feelings of despair, and isolation from family members were identifies as psychological factors and barriers to diabetes self‐management and adherence to a healthy mode of living.^16^, ^25^, ^26^, ^29^ Also, one study reported that lifelong self‐care management and the level of acceptance of strict adherence to the regulations lead to a considerable mental burden in old‐age women with diabetes.^16^
  1.4 Financial factors
    The common factors associated with financial factors were financial constraints,^15^, ^17^, ^22^, ^23^, ^26^, ^29^ cost of food and medications,^17^, ^21^, ^25^ poverty,^14^, ^18^, ^26^, ^27^ transportation costs,^15^, ^18^, ^23^ and family size.^18^ Those with financial issues were less likely to follow up on their treatment process. Two studies from the United States reported the cost of medication^25^ and a lack of financial resources to visit a physician or buy medicines as a barrier.^29^ Cécile Leyns et al. showed despite affordable transportation to healthcare facilities, transportation for urgent medical assistance, such as an ambulance was unavailable in a Peri‐urban in Bolivia.^20^ Indian studies reported due to financial constraints, diabetic patients in India did not take the medication regularly, changed their treatment due to inaccessibility, transportation barrier, or money constraints in India and had to reduce their medication dosage or even some preferred modern systems of medicine.^22^, ^23^ GPs believed that financial incentives could encourage low socioeconomic position patients to participate in diabetes management.^27^ Transport improvement could positively influence disease management among people living in an urban diabetogenic area.^15^
2. Healthcare system factors
  2.1 Patient's education processes
    Dissatisfaction with the presented information,^15^ poor information about available services,^21^ educational barriers (unknowing of complications of asymptomatic diseases),^26^ and availability of poor information for family members^29^ was a barrier while creating educational materials tailored to patients' literacy level^27^, providing regular educational courses,^26^ diversity of education (e.g., in‐person/recorded verbal education, receiving pamphlets),^26^ access to informal medical information,^14^ narrative information and practical programs,^15^ providing/presenting information in the community^15^ were perceived as a positive facilitator for disease management. The perceived skills of healthcare providers and traditional knowledge and beliefs of the patients played a substantial role in whether a patient will seek medical help for their symptoms.^19^
  2.2 Financial protection
    A study found that lacking health insurance or inadequate insurance coverage tended to appear as barriers to patients' diabetes care and reported patients with private insurance complained about costly co‐pays and premiums.^14^ Cuesta‐Briand et al. reported although patients in Perth city in Australia did not incur any out‐of‐pocket expenses to visit a GP, the cost was commonly reported as a barrier to accessing healthcare services.^21^ A study reported that to cope with financial constraints, financial protection measures should be complemented and integrated with broader social protective measures to promote livelihoods and other social services.^22^ As a facilitator for slum‐dwellers, financial and appropriate coverage of diabetes costs by insurance organizations can lead to better self‐management and reduce the cost of diabetes management.^20^, ^24^, ^27^
  2.3 Service delivery
    Regarding service delivery, the studies reported the importance of accessibility, availability, quality of care, and patient‐provider relationships.^14^, ^15^, ^17^, ^18^, ^19^, ^20^, ^21^, ^22^, ^23^, ^24^, ^25^, ^26^, ^27^, ^29^, ^30^ Patient‐provider/physician relationship was commonly found to influence disease management.^14^, ^15^, ^24^, ^25^, ^29^, ^30^ Poor communication with providers may hinder medication adherence.^25^ Communication barriers between the providers, patients, and organization, and difficulty accessing primary care often results in patients forgoing needed care.^24^ Fragmented healthcare delivery was reported as a barrier to continuing treatment in India.^22^ The healthcare professional can help diabetic patients to overcome the barriers to self‐management, such as lack of family support, negative perception of time, and so on.^16^ An Australian study showed that when healthcare professionals personalize care, develop rapport and express empathy, more effective care can be delivered.^15^
3. Contextual factors
  The most important themes related to contextual factors were: (1) family support, (2) social support, and (3) environmental support.
  3.1 Family support
    This included family size, generation gap, gender discrimination, information, reminding to take medicine, and psychological factors.^14^, ^16^, ^17^, ^18^, ^20^, ^21^, ^22^, ^23^, ^24^, ^25^, ^28^, ^29^, ^35^ Several studies reported the important role of family members, particularly spouses and children, in diabetes self‐management, and also were seen as a facilitator to adherence/diet and a source of information.^14^, ^17^, ^26^, ^28^, ^29^ A qualitative study found that although family support could positively influence diabetes self‐management among patients, the fear of becoming a burden on the family may limit these positive impacts.^14^ Being dependent on family members to obtain medications or having responsibilities to the family interfered with diabetes self‐management, and some patients felt isolated from their family members.^29^ A study reported cultural beliefs and values could influence the perceived feelings of despair and isolation from family members, while another study reported that culture did not seem to have an impact on the attitudes and behaviors to diabetes self‐management.^35^
  3.2 Social support
    These included social isolation, stigma, and peer groups. Four studies^14^, ^15^, ^17^, ^25^ described that patients might be isolated from society because of the nature of the disease, such as the use of insulin and the stigma surrounding the illness. Social support and the creation of peer groups to exchange information can help patients provide dietary and exercise programs and empower them regarding psychology.^17^, ^24^ Social support from families, health workers, and diabetes peer groups can facilitate healthy eating and physical activity.^17^ A study reported patients with T2D had both negative and positive perceptions regarding overweight, such as stigma, health problems, and negative effects on personal relationships, but obesity was also seen as a sign of happiness, wealth, and a genetic origin.^19^ Social support networks can mediate the impacts of economic and environmental disadvantages by improving access to social capital.^14^
  3.3 Environmental factors
    Regarding “environmental factors,”^15^, ^17^, ^19^, ^20^, ^30^ the authors recognized the importance of neighborhood safety and a supportive environment to disease management. Unsafe environments to exercise and go to health centers were complaints by slum‐dwellers patients with T2D.^15^ Barriers to physical activity could be related to a lack of willingness and resources to exercise and a social upbringing, especially where there was no opportunity to exercise.^19^

**Table 3:** Factors affecting diabetes management among slum‐dwellers.

Analytical themes	Descriptive themes	Codes
1. Individual factors	1.1 Lifestyle behavior	Change resistance Comorbidities Nature of the disease Lifestyle adherence Treatment adherence
	1.2 Informational factors	Literacy Knowledge about diabetes and its complications Healthy lifestyle knowledge Health beliefs
	1.3 Psychological factors	Fear Depression Denial
	1.4 Financial factors	Affordability to healthy diet adherence Medication costs Transport costs Family size Practical dietary
2. Healthcare system factors	2.1 Patient's education processes	Conflicting information Vague information Educational materials
	2.2 Financial protection	Health insurance coverage Out‐of‐pocket payments Co‐payment
	2.3 Service delivery	Accessibility Availability Quality of care Patients–provider communication
3. Contextual factors	3.1 Family support	Family size Generation gap/inter‐generational conflicts Gender discrimination Informational support Reminding to take medicine Psychological
	3.2 Social support	Social isolation Stigma Peer‐groups
	3.3 Environmental factors	Neighborhood safety Supportive environment

## 4 DISCUSSION

This study aimed to identify barriers and facilitators to T2D management in slum residents by synthesizing qualitative studies. We found 17 studies on the facilitators and barriers to T2D management among slum‐dwellers. We identified three analytical themes, including individual factors, contextual factors, and health system‐related factors to T2D management in slum‐dwellers.

We identified several factors that are particularly pertinent in slum settings, such as difficulty in dietary adherence, adherence medication,^15^, ^16^, ^17^, ^18^, ^26^ lifestyle changes/behavior,^15^, ^16^, ^18^, ^22^, ^23^, ^26^ literacy and knowledge about the disease and diet,^14^, ^15^, ^17^, ^18^, ^19^, ^26^, ^30^, ^35^ costs and financial issues,^14^, ^17^, ^18^, ^19^, ^21^, ^22^, ^23^, ^24^, ^25^, ^27^, ^29^, ^35^ family/social/friends support,^14^, ^25^, ^26^, ^29^ availability/accessibility of healthcare services, the costs of health services,^15^, ^17^, ^18^, ^19^, ^20^, ^23^ patients‐providers interaction.^14^, ^24^, ^25^, ^29^, ^30^ In line with our study, a previous scoping review suggested that factors such as knowledge, perception (including misconception and distrust), financial issues, stigma, healthcare needs and health services, competing priorities and inadequacy of social support, and so on, in the existing health system all contribute to the challenges faced by slum‐dwellers.^39^

Costs and financial issues were frequently reported in studies as factors affecting healthcare‐seeking behaviors, disease management, and also as a barrier to dietary adherence and eating healthy food, adherence to medication, and diabetes self‐management.^14^, ^17^, ^18^, ^19^, ^21^, ^22^, ^23^, ^24^, ^25^, ^27^, ^29^, ^35^ These barriers were also reported in previous studies.^40^, ^41^, ^42^ A study in Iran reported financial issues being the main reason for not taking prescribed drugs.^43^ According to a cross‐sectional study in Iran, 45% of patients were forced to forgo treatment because of financial barriers and treatment quality discontent.^44^ It seems that the measures to provide financial protection for health to this group need to be complemented and integrated with broader social protective measures that promote livelihoods and other social services.^22^

Literacy and knowledge about the disease, how to diet, or how to eat were frequently reported in studies as both facilitators and barriers.^14^, ^15^, ^17^, ^18^, ^19^, ^20^, ^24^, ^26^, ^29^, ^30^, ^35^ Health literacy was found to be poorer among older people, minority groups, and people from lower socioeconomic circumstances.^45^ Living in slums has been found to be associated with poor knowledge about the cause and preventability of diseases^46^ and also a barrier to accessing healthcare services.^47^, ^48^ Poor health literacy and knowledge can limit patients' ability to care for their medical problems.^35^ Educational interventions can reduce diabetes incidence by 54% through a reduction in fasting blood glucose, body mass index, and waist circumference. Regardless of educational intervention duration, the diabetes risk parameters may improve at as low as 6 months.^49^ Some identified facilitators in studies were education in a narrative form,^15^ providing education and support within the family context, community settings, and social groups,^29^ delivering pamphlets,^26^ receiving education from healthcare providers^20^ and trained facilitators (e.g., retired nurses),^19^ availability of different education modalities,^26^ culturally and linguistically appropriate education and intervention,^29^, ^30^ and community‐based and family‐centered education.^30^ More community‐based education regarding T2D self‐management and treatment, with peer group education and the use of trained facilitators, were seen as facilitators.^19^

Socio‐environmental, cultural factor,^17^ and patients' perceptions regarding the disease can impede lifestyle behaviors.^18^ The available literature demonstrated that lifestyle changes with physical activity could alter the incidence of diabetes or one of the T2D risk factors.^50^ Reasons causing patients with T2D in slum areas to stop physical activity were fear of public ridicule, comorbidities,^17^ laziness,^19^ fear of walking in the street,^15^ and maintaining this as a habit is a tricky part of life as a habit.^16^ A facilitator that can positively affect lifestyle changes/behaviors is how to educate patients. Narrative communication can significantly influence diet and exercise.^51^ Also, diabetes peer groups can act as a social support network and facilitator for diet and physical activity, especially in urban areas.^17^ Family support, emphasis from the health worker, household chores, and type of work were identified facilitators of physical activity.^17^

A factor influencing T2D management among those living in slums was the nature of the disease. Diabetes self‐management can be influenced by complex traditional and religious beliefs, social norms, and peer pressure.^19^ A study in South Africa found that diabetes was interpreted as a physically and emotionally dangerous disease caused by sociocultural‐related factors and significantly influenced by the patients' food culture and traditional beliefs. A study in Cameroon showed culture could strongly influence patients' definition of diabetes.^52^

Family support can play a crucial role in diabetes self‐management.^29^ Family members can act as a support system for medication adherence and as a health information source.^25^, ^53^, ^54^ One reason that causes family members don't support patients with diabetes is their lack of knowledge about the disease.^29^ Jackson et al. reported that while family, culture, and religion were strong positive influences on health behaviors, poor health‐care access, and patient‐provider interactions adversely impacted self‐care ability.^24^

Slum‐dwellers frequently reported a lack of health services and insufficient availability and accessibility of healthcare services as barriers.^17^, ^20^, ^25^ Health systems can assist patients with diabetes by providing timely, appropriate, and satisfactory services. Previous studies reported lack of time and competing priorities as factors affecting healthcare‐seeking behavior^55^, ^56^, ^57^ and health service utilization.^58^, ^59^, ^60^ A scoping review demonstrated that slum residents have to strike when making decisions on healthcare seeking and utilization,^39^ lack of health insurance coverage, expensive co‐payments, and mistrust of the medical system is a barrier for low‐income populations with diabetes.^14^, ^61^ Poor information, lack of skills, poor communication skills, and high workload in delivering care to patients by healthcare systems lead to poor quality of care and poor T2D management among slum‐dwellers.^62^ In Pakistan, due to ignorance and nonavailability of examination and screening, less population of slum‐dweller with diabetes was aware of their disease.^63^ A study in Iran showed that T2D patients do not adhere to the formal structure of the health system because of systemic difficulty.^64^ The cost and the availability of services delivered by health systems can influence the proper diagnosis and treatment of diabetes in slum‐dwellers.^65^ Previous studies have reported that healthcare cost is a major barrier to seeking healthcare and accessing and utilizing services.^21^, ^66^, ^67^ It is important to provide financial protection to patients against a huge impoverishing out‐of‐pocket healthcare costs.^22^

Poor patient‐provider interaction was reported in studies as a barrier to disease management.^15^, ^25^, ^29^, ^30^ An important facilitator of adherence is effective interaction with providers.^25^ Earlier studies demonstrated that poor patient‐provider communication results in uncertainty about diagnosis^68^ and negatively affects self‐care.^36^, ^68^, ^69^ A study showed that dissatisfaction and mistrust of healthcare providers were higher among those with lower health literacy and indigenous peoples who have experienced stigma, marginalization, and discrimination in the healthcare system.^70^ Building trust and enhancing communication between patients and providers to create plans of care together can be considered a facilitator.^30^, ^71^ A patient's ability to self‐manage diabetes is profoundly influenced by interweaving social support structures with the doctor–patient relationship and the interaction with the healthcare system.^14^ Previous studies among non‐Latino populations found that effective patient‐provider communication and offering essential skills for healthcare providers can facilitate adherence,^25^, ^72^, ^73^ and primary care providers' understanding of the context of patients' social support is vital to improving their health.^14^

After completing the meta‐synthesis, we compared studies with high and moderate quality in terms of relative contributions to analytical themes but found no difference.

## 5 STRENGTHS AND LIMITATIONS

Our study has strengths and limitations that deserve to be mentioned. Its main strength is the provision of comprehensive information about determinants of the management of T2D among slum‐dwellers. With respect to limitations, we included only studies that were written in English, whereas slum‐dwelling is a common phenomenon in LMICs. So, the findings may not be applicable to other regions. Due to the insufficient sample size and sampling methods in qualitative research, the results may not be generalizable.

## 6 CONCLUSIONS

Diabetes is a multifactorial, chronic, and complex disease. In addition to individual barriers, many systemic barriers caused by the person's living environment play a major role in the failure to manage this disease. It is important to conduct interventions at the level of the individual, the health system, and the context to effectively manage diabetes in slums. This requires interaction and cooperation between the patient, his/her family, and the healthcare system. In addition, fundamental reforms in a person's living environment are necessary to improve the effectiveness of interventions at the individual, patient's family, and health system levels. Therefore, policy‐making for diabetes management in the slum areas requires the participation and cooperation of other relevant stakeholders, most of whom span sectors outside the health sector. It is necessary to define interventions at different levels separately and implement them in a coordinated and aligned manner. Although focusing on empowering the patient and his/her family in the short term can partially modulate the negative consequences of other environmental and systemic factors, in the long term, fundamental reforms are necessary by a focus on optimizing the living environment, eliminating social‐economic inequalities, and strengthening the infrastructure of the health system, increasing financial support for health centers in slums areas and improving the quality of services provided in these areas aiming at increasing the access and benefit of these groups from health services. Finally, due to the special conditions of slum areas, it is suggested that diabetes policy and planning in these areas ought to be of great interest.

## AUTHOR CONTRIBUTIONS

**Fawzieh Ghammari**: Conceptualization; formal analysis; methodology; writing—original draft. **Habib Jalilian**: Conceptualization; formal analysis; methodology. **Rahim Khodayari‐Zarnaq**: Formal analysis; writing—review and editing. **Masumeh Gholizadeh**: Formal analysis; supervision; writing—review and editing.

## CONFLICT OF INTEREST STATEMENT

The authors declare no conflict of interest.

## ETHICS STATEMENT

This study was part of the PhD thesis of F. G. and was approved by the Ethics Committee of Tabriz University of Medical Sciences (Reference No: IR.TBZMED.REC.1400.961). IR. TBZMED. REC.1400.961).

## TRANSPARENCY STATEMENT

The lead author Masumeh Gholizadeh affirms that this manuscript is an honest, accurate, and transparent account of the study being reported; that no important aspects of the study have been omitted; and that any discrepancies from the study as planned (and, if relevant, registered) have been explained.

## Supporting information

Supporting information.

Supporting information.

## Data Availability

The data that support the findings of this study are available from the corresponding author upon reasonable request.
