# Supplementary material for: Barriers and facilitators to type 2 diabetes management among slum‐dwellers: A systematic review and qualitative meta‐synthesis
Source: Health Sci Rep. 2023 Apr 27;6(5):e1231. doi: 10.1002/hsr2.1231 (PMC10140644; doi:10.1002/hsr2.1231)
Supplement: Supplementary file 1 — Supporting information. [file HSR2-6-e1231-s002.docx]

**Applied search strategy in Scopus**

TITLE-ABS-KEY("Patient acceptance of health care" OR "Health Care Utilization" OR "Utilization, Health Care" OR Adherence OR “non-adherence” OR Compliance OR “non-compliance” OR Concordance OR "Patient Acceptance of Healthcare" OR "Healthcare Patient Acceptance" OR "Healthcare Patient Acceptances" OR "Nonacceptors of Health Care" OR "Care Nonacceptor, Health" OR "Care Nonacceptors, Health" OR "Health Care Nonacceptor" OR "Health Care Nonacceptors" OR "Health Care Seeking Behavior" OR "Acceptors of Health Care" OR "Care Acceptor, Health" OR "Health Care Acceptors" OR "Acceptability of Health Care" OR "Health Care Acceptability" OR "Acceptability of Healthcare" OR "Healthcare Acceptabilities" OR "Treatment-seeking" OR "Health Care seeking" OR "Healthcare seeking" OR "Information seeking behavior" OR "health-seeking behaviors" OR "Diabetes seeking care" OR "Support-seeking" OR "Care-Seeking" OR "Service Seeking" OR "Help-seeking" OR "Seeking health care" OR "Seeking health services" OR "Seeking health advice" OR "Seeking health treatment" OR "Seeking Health Information" OR “unmet need”) AND TITLE-ABS-KEY("diabetes mellitus, type 2" OR ”Diabetes mellitus” OR NIDDM OR diabetic OR MODY OR "Maturity Onset Diabetes" OR “Maturity-Onset Diabetes " OR “Diabetes, maturity-onset” OR “Type 2 Diabetes" OR "Diabetes, Type 2") AND TITLE-ABS-KEY(slums OR "peri-urban" OR suburb OR Colonia OR outskirts OR "informal settlement" OR "neighborhood disadvantage" OR “socioeconomic disadvantage” OR "Urban poor settlement" OR "Semi-urban" OR "Sub-urban")

**Applied search strategy in web of sciences**

TS=("Patient acceptance of health care" OR "Health Care Utilization" OR "Utilization, Health Care" OR Adherence OR “non-adherence” OR Compliance OR “non-compliance” OR Concordance OR "Patient Acceptance of Healthcare" OR "Healthcare Patient Acceptance" OR "Healthcare Patient Acceptances" OR "Nonacceptors of Health Care" OR "Care Nonacceptor, Health" OR "Care Nonacceptors, Health" OR "Health Care Nonacceptor" OR "Health Care Nonacceptors" OR "Health Care Seeking Behavior" OR "Acceptors of Health Care" OR "Care Acceptor, Health" OR "Health Care Acceptors" OR "Acceptability of Health Care" OR "Health Care Acceptability" OR "Acceptability of Healthcare" OR "Healthcare Acceptabilities" OR "unmet need" OR "Treatment-seeking" OR "Health Care seeking" OR "Healthcare seeking" OR "Information seeking behavior" OR "health-seeking behaviors" OR "Diabetes seeking care" OR "Support-seeking" OR "Care-Seeking" OR "Service Seeking" OR "Help-seeking" OR "Seeking health care" OR "Seeking health services" OR "Seeking health advice" OR "Seeking health treatment" OR "Seeking Health Information") AND TS=("diabetes mellitus, type 2" OR diabetic OR NIDDM OR "Maturity-Onset Diabetes " OR "Diabetes, maturity-onset" OR MODY OR "Maturity Onset Diabetes" OR "Type 2 Diabetes" OR "Diabetes, Type 2") AND TS=(slums OR "peri-urban" OR suburb OR Colonia OR outskirts OR "informal settlement" OR "neighborhood disadvantage" OR "Urban poor settlement" OR "Semi-urban" OR "Sub-urban" OR "socioeconomic disadvantage")

**Applied search strategy in PubMed**

((((((((((((((((((((((((((((((((((((((((((((((((((((((((“patient acceptance of health care”[MeSH Terms]) OR (“Treatment-seeking”[Title/Abstract])) OR (management[Title/Abstract])) OR (“Health Care seeking”[Title/Abstract])) OR (“Healthcare seeking”[Title/Abstract])) OR (Adherence[Title/Abstract])) OR (“non-adherence” [Title/Abstract])) OR (Compliance[Title/Abstract])) OR (“non-compliance” [Title/Abstract])) OR (Concordance[Title/Abstract])) OR (“Information seeking behavior”[Title/Abstract])) OR (“Health-seeking behaviors”[Title/Abstract])) OR (“Diabetes seeking care”[Title/Abstract])) OR (“Support-seeking”[Title/Abstract])) OR (“Care-Seeking”[Title/Abstract])) OR (“Service Seeking”[Title/Abstract])) OR (“Help-seeking”[Title/Abstract])) OR (“Seeking health care”[Title/Abstract])) OR (“Seeking health services”[Title/Abstract])) OR (“Seeking Health Services”[Title/Abstract])) OR (“Seeking health advice”[Title/Abstract])) OR (“unmet need”[Title/Abstract])) OR (“Seeking health treatment”[Title/Abstract])) OR (“Seeking Health Information”[Title/Abstract])) AND (“diabetes mellitus, type 2”[MeSH Terms])) OR (“diabetes mellitus, type 2”[Title/Abstract])) OR (diabetic[Title/Abstract])) OR (MODY[Title/Abstract])) OR (NIDDM[Title/Abstract])) OR (“Maturity Onset Diabetes”[Title/Abstract])) OR (“Type 2 Diabetes”[Title/Abstract])) OR (“Diabetes, Type 2”[Title/Abstract])) AND (slums[Title/Abstract])) OR (“peri-urban”[Title/Abstract])) OR (suburb[Title/Abstract])) OR (Colonia[Title/Abstract])) OR (outskirts[Title/Abstract])) OR (“informal settlement”[Title/Abstract])) OR (“neighborhood disadvantage”[Title/Abstract])) OR (“Urban poor settlement”[Title/Abstract])) OR (“Semi-urban”[Title/Abstract])) OR (“Sub-urban”[Title/Abstract]) OR (“Disadvantage socioeconomic”[Title/Abstract]))

5863 results
