# Supplementary material for: Barriers and facilitators to type 2 diabetes management among slum‐dwellers: A systematic review and qualitative meta‐synthesis
Source: Health Sci Rep. 2023 Apr 27;6(5):e1231. doi: 10.1002/hsr2.1231 (PMC10140644; doi:10.1002/hsr2.1231)
Supplement: Supplementary file 2 — Supporting information. [file HSR2-6-e1231-s001.docx]

Quality Assessment Tool (QATSDD) scores for all reviewed papers (scores range from 0 – 3)

| Citation number | Item 1 | Item 2 | Item  3 | Item 4 | Item 5 | Item 6 | Item 7 | Item 8 | Item 9 | Item 10 | Item 11 | Item 12 | Item 13 | Item 14 | Item 15 | Item 16 | score | % |
| --- | --- | --- | --- | --- | --- | --- | --- | --- | --- | --- | --- | --- | --- | --- | --- | --- | --- | --- |
| 23 | 3 | 3 | 3 | 3 | 2 | 3 | 2 | 3 | N/A | N/A | 3 | N/A | 3 | 0 | 0 | 2 | 30/39 | 76.9% |
| 24 | 3 | 3 | 3 | 2 | 3 | 3 | 2 | 3 | N/A | N/A | 3 | N/A | 3 | 0 | 1 | 3 | 32/39 | 82% |
| 20 | 2 | 3 | 3 | 3 | 1 | 3 | 3 | 3 | N/A | N/A | 3 | N/A | 3 | 3 | 1 | 1 | 32/39 | 82% |
| 26 | 3 | 3 | 2 | 3 | 2 | 3 | 3 | 3 | N/A | N/A | 3 | N/A | 3 | 0 | 0 | 0 | 28/39 | 71.7% |
| 32 | 3 | 3 | 3 | 3 | 2 | 3 | 2 | 3 | N/A | N/A | 3 | N/A | 3 | 3 | 1 | 2 | 34/39 | 87.1% |
| 18 | 3 | 3 | 3 | 1 | 2 | 3 | 2 | 2 | N/A | N/A | 3 | N/A | 3 | 3 | 0 | 2 | 30/39 | 76.9% |
| 28 | 3 | 3 | 1 | 3 | 0 | 3 | 3 | 3 | N/A | N/A | 3 | N/A | 3 | 0 | 0 | 0 | 25/39 | 64.1% |
| 31 | 3 | 3 | 3 | 3 | 2 | 3 | 3 | 3 | N/A | N/A | 3 | N/A | 3 | 0 | 0 | 2 | 31/39 | 79.4% |
| 27 | 3 | 3 | 3 | 3 | 1 | 3 | 1 | 3 | N/A | N/A | 3 | N/A | 3 | 0 | 0 | 2 | 28/39 | 71.7% |
| 30 | 3 | 3 | 2 | 3 | 2 | 2 | 3 | 3 | 0 | 3 | 3 | 3 | 3 | 0 | 0 | 0 | 33/48 | 68.7% |
| 29 | 3 | 3 | 3 | 3 | 0 | 3 | 3 | 3 | 1 | 3 | 3 | 3 | 3 | 0 | 0 | 2 | 36/48 | 75% |
| 25 | 3 | 3 | 3 | 2 | 0 | 3 | 2 | 3 | N/A | N/A | 3 | N/A | 3 | 0 | 0 | 2 | 27/39 | 69.2% |
| 16 | 3 | 3 | 3 | 2 | 0 | 3 | 3 | 3 | N/A | N/A | 3 | N/A | 3 | 0 | 0 | 3 | 29/39 | 74.3% |
| 21 | 3 | 3 | 1 | 2 | 2 | 3 | 2 | 3 | N/A | N/A | 3 | N/A | 3 | 3 | 0 | 1 | 29/39 | 74.3% |
| 19 | 3 | 3 | 1 | 1 | 1 | 3 | 3 | 3 | N/A | N/A | 3 | N/A | 2 | 0 | 0 | 1 | 24/39 | 61.5% |
| 17 | 3 | 3 | 3 | 3 | 0 | 3 | 3 | 3 | N/A | N/A | 3 | N/A | 3 | 3 | 0 | 3 | 33/39 | 84.6% |
| 22 | 3 | 3 | 3 | 1 | 0 | 3 | 2 | 2 | N/A | N/A | 3 | N/A | 3 | 0 | 1 | 2 | 26/39 | 66.6% |

Item 1: Explicit theoretical framework
Item 2: Statement of aims/objectives in main report
Item 3: Clear description of research setting
Item 4: Evidence of sample size considered in terms of analysis
Item 5: Representative sample of target group of a reasonable size
Item 6: Description of procedure for data collection
Item 7: Rationale for choice of data collection tool(s)
Item 8: Detailed recruitment data
Item 9: Statistical assessment of reliability and validity of measurement tool(s) (Quantitative studies only)
Item 10: Fit between research question and method of data collection (Quantitative studies only)
Item 11: Fit between research question and format and content of data collection tool e.g. interview schedule (Qualitative studies only)
Item 12: Fit between research question and method of analysis (Quantitative studies only)
Item 13: Good justification for analytic method selected
Item 14: Assessment of reliability of analytic process (Qualitative studies only)
Item 15: Evidence of user involvement in design
Item 16: Strengths and limitations critically discussed
